# An epidemiologic comparison of acute and overuse injuries in high school sports

**DOI:** 10.1186/s40621-021-00344-8

**Published:** 2021-08-11

**Authors:** Erin E. Ritzer, Jingzhen Yang, Sandhya Kistamgari, Christy L. Collins, Gary A. Smith

**Affiliations:** 1 Center for Injury Research and Policy, Abigail Wexner Research Institute at Nationwide Children’s Hospital, Columbus, OH USA; 2 Lake Erie College of Osteopathic Medicine, 5000 Lakewood Ranch Blvd, FL 34211 Bradenton, USA; 3 Department of Pediatrics, The Ohio State University College of Medicine, Columbus, OH USA; 4 Datalys Center for Sports Injury Research and Prevention, 401 West Michigan Street, Suite 500, Indianapolis, IN USA; 5 Child Injury Prevention Alliance, Columbus, OH USA

**Keywords:** High School Athletes, Gender-Comparable Sports, Injury Reporting

## Abstract

**Background:**

Acute and overuse injuries affect millions of high school athletes annually and a better understanding of differences between these injuries is needed to help guide prevention, treatment, and rehabilitation strategies. This study compares acute and overuse injuries using a nationally representative sample of high school athletes.

**Methods:**

Injuries among United States high school athletes participating in 5 boys’ sports (football, soccer, basketball, wrestling, baseball) and 4 girls’ sports (soccer, volleyball, basketball, softball) reported in the High School RIO™ surveillance system during the 2006-07 through 2018-19 school years were classified as acute or overuse. National estimates and injury rates were calculated.

**Results:**

Of 17 434 646 estimated injuries, 92.0 % were acute and 8.0 % were overuse. The acute injury rate was higher than the overuse injury rate among both male (Rate Ratio [RR] 16.38, 95 % CI: 15.70–17.10) and female (RR 8.14, 95 % CI: 7.71–8.60) athletes. The overuse injury rate per 10,000 athlete exposures among female athletes (1.8) was slightly higher than among males (1.4). The rate of acute injury compared with the rate of overuse injury was higher during competition (RR 32.00, 95 % CI: 29.93–34.22) than practice (RR 7.19, 95 % CI: 6.91–7.47). Boys’ football contributed the most acute (42.1 %) and overuse (23.7 %) injuries among the 9 sports. Among female sports, girls’ soccer contributed the most acute (15.6 % of all acute injuries) and overuse (19.4 % of all overuse injuries) injuries. The lower extremity was most commonly injured in acute (48.9 %) and overuse (65.9 %) injuries. Ligament sprain (31.7 %) and concussion (21.0 %) were the most common acute injury diagnoses, while muscle strain (23.3 %) and tendonitis (23.2 %) were the most common overuse injury diagnoses. Compared with acute injuries, overuse injuries were more likely to result in time loss from sports participation of < 1 week among both boys and girls and across most sports. Acute injuries were more likely than overuse injuries to cause a time loss of 1–3 weeks or medical disqualification from sports participation.

**Conclusions:**

Acute and overuse injuries display many differences that provide opportunities for data-informed athlete preparation, treatment, and rehabilitation, which may reduce injuries and improve injury outcomes in high school athletics.

## Background

Approximately 7.9 million athletes participated in high school sanctioned sports during the 2018–2019 school year, which is the third highest annual number of participants reported since the first National Federation of State High School Associations survey was implemented in 1971 (Participation 2019). Youth sports provide many benefits, including opportunities to improve social skills, self-esteem, and general physical fitness; yet sports participation also has drawbacks, including exposure to stressful situations and the risk of injury (Merkel and Merkel 2013). It has been well established that injuries during developmental years can have lasting effects on the health of athletes. With participation numbers rising, the consequences of injury, both immediate and long-term, will continue to place a significant burden on the healthcare system.

Using data collected from various injury reporting systems, existing studies have analyzed sports-related injuries at individual universities, groups of high schools, or within specific cohorts of athletes, which limit their ability to establish patterns that can be generalized across larger populations (Cuff et al. 2010; Liller et al. 2019; Lundberg Zachrisson et al. 2020; Yang et al. 2012). Other studies have been limited to a certain sport, type of injury, or body part, also reducing their generalizability (Badgeley et al. 2013; Ingram et al. 2008; Kerr et al. 2011; Saper et al. 2018; Swenson et al. 2010; Xiang et al. 2014; Yard et al. 2008; Yard et al. 2008). High School Reporting Information Online (RIO™) is currently the largest sports-related injury surveillance system that uses a nationally representative sample of United States (US) high school athletes. While it has been used in many peer-reviewed publications, few have studied overuse injuries (Cuff et al. 2010; Kerr et al. 2018; Rechel et al. 2008; Schroeder et al. 2015). To-date, no studies have directly compared characteristics of acute and overuse injuries using a nationally representative database.

This study compared acute and overuse injuries using a nationally representative sample of US high school athletes participating in 5 boys’ sports (football, soccer, basketball, wrestling, baseball) and 4 girls’ sports (soccer, volleyball, basketball, softball) using data obtained from RIO™. Specifically, this study investigated differences in frequency and rates of injury as well as injury characteristics (i.e., body site, diagnosis, recurrence, and time loss from sports participation) between acute and overuse injuries while also considering the role that gender plays in these differences. Study findings fill a gap in our knowledge about these injuries that will help guide prevention, treatment, and rehabilitation strategies.

## Methods

### Source of Data

Study data were obtained from the national high school sports-related injury surveillance system, RIO™. RIO™ data collection methods have been detailed previously (Kerr et al. 2018; Rechel et al. 2008). Briefly, starting in the 2005-06 school year, high schools with at least one certified athletic trainer (AT) affiliated with the National Athletic Trainers’ Association were invited to participate in the surveillance system. One-hundred high schools were chosen randomly from 8 strata, based on geographic region and school size, to form a nationally representative sample of high school athletes participating in 9 common high school sports; the sample has been updated annually. RIO™ also includes data on injuries and exposures from additional sports based on convenience sampling; these injuries were not included in this study because they are unable to be used to calculate national estimates. A weighting algorithm was used to generate national injury estimates for the 9 sports included in this study. ATs from participating high schools reported injuries and athlete exposures (AEs) weekly to RIO™. For each injury, ATs reported details on injured athletes’ demographics, sport, injury circumstances including whether it was a new or recurrent injury, mechanisms of onset, preliminary diagnosis, treatment(s) received, and return to play/medical disqualification information. Medical disqualification is defined as discontinuation of an athlete’s participation for a season or a career within the sport in which the injury occurred. ATs were able to update injury reports within the database if new information became available, including, when appropriate, updated diagnoses from physicians or additional details about the injury.

### Definitions of Injury and Athlete Exposure

In this study, an injury satisfied all these criteria: (1) occurred due to participation in an organized high school practice or competition, (2) required medical attention, and (3) resulted in a time loss from participation of at least 1 day beyond the day the injury occurred, or any fracture, concussion, or dental injury, regardless of whether it restricted an athlete’s participation. An AE was defined as one athlete participating in one practice or one competition.

### Study Population and Gender-Comparable Sports

This study included athletes injured while participating in 9 sports (boys’ football, boys’ and girls’ soccer, girls’ volleyball, boys’ and girls’ basketball, boys’ wrestling, boys’ baseball, and girls’ softball) from the 2006-07 through 2018-19 school years. Data from the 2005-06 school year were not included because the database did not include “overuse/chronic” as an option for a basic injury mechanism during that year. Six sports were considered gender-comparable based on similar rules or play tactics: boys’ and girls’ soccer, boys’ and girls’ basketball, and boys’ baseball and girls’ softball (Shanley et al. 2011).

### Categorization of Acute and Overuse Injuries

**Fig. 1:**
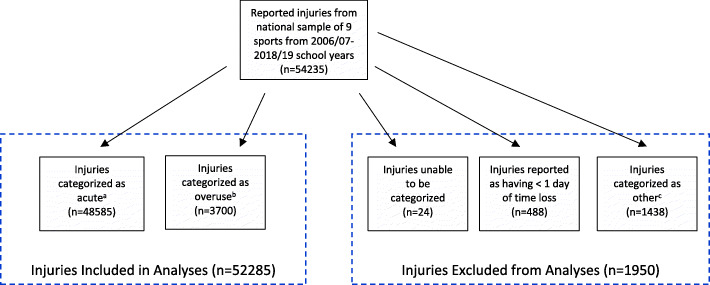
Flowchart of Classification of Injuries. ^a^The following types of injuries were categorized as acute injuries: abrasion; concussion; contusion; dislocation; fracture; hemorrhage; hernia; hyperextension; internal injury (non-hemorrhage); laceration; ligament sprain (complete tear, incomplete tear, severity unknown); muscle strain (complete tear, incomplete tear, severity unknown); tendon strain (complete tear, incomplete tear, severity unknown); nerve injury; separation; torn cartilage; avulsion; multiple injuries; and subluxation. However, if injuries of these types were reported to be due to an overuse/chronic or illness mechanism, then they were not included in the acute category; they were categorized as detailed below. ^b^The following types of injuries were categorized as overuse injuries: blister; bursitis; inflammation; stress fracture; tendonitis; apophysitis; plantar fasciitis; shin splints; and spondylolysis AND/OR any injury reported to be due to an overuse/chronic mechanism. ^c^The following types of injuries were categorized as other injuries: burn; dehydration; heatstroke/heat; infection (including skin infection); frostbite/cold related; cardiac event (acute); chronic disease/trait; respiratory event; heat illness/injury; and mental health concern AND/OR any injury reported to be due to an illness, other, or unknown mechanism

Injuries were categorized as acute or overuse using the algorithm outlined in the flowchart in Fig. 1. Overuse injury has been previously characterized by (1) a mechanism of gradual onset, and (2) an underlying pathogenesis of repetitive microtrauma (Roos et al. 2014). In the current study, overuse injuries included those resulting from repetitive exposure or an overuse/chronic mechanism, and acute injuries included those caused immediately by a specific event. Injuries were first categorized as overuse or acute based on reported injury diagnosis. Any injury that was reported by an AT as being due to an overuse/chronic mechanism was categorized as overuse. If an injury had a diagnosis that initially was considered to be in the acute injury category but was reported to have an overuse/chronic mechanism, then it was recategorized as an overuse injury.

### Categorization of Injuries by Body Site and Diagnosis

The injured body part was grouped into 5 categories: (1) head/face/neck, (2) trunk, (3) upper extremity, (4) lower extremity, and (5) other. The “other” body site category included injuries to male genitalia and body parts listed as “other” in RIO™. Injury diagnoses were grouped by their overall diagnosis. For example, injuries coded as ligament sprain, ligament sprain (complete tear), ligament sprain (incomplete tear), and ligament sprain (unknown severity) were grouped as ligament sprain (any severity). “Other unspecified” was used to describe an injury diagnosis reported by ATs as “other,” which prompted the ATs to provide more information for an unlisted or unusual diagnosis in RIO™. The “other specified” category included all diagnoses that were selectable at data entry but were not among the top 10 most common diagnoses.

### Statistical Analysis

Data were analyzed using IBM SPSS Statistics for Windows, Version 26 (Armonk, NY: IBM Corp) and SAS 9.4 (SAS Institute, Inc, Cary, NC). Sampling weights provided by RIO™ were used to calculate national estimates. Descriptive statistics included national estimates and proportions with 95 % confidence intervals (CIs). Poisson regression was used to estimate the rate ratio (RR) using the rate of overuse injury as the denominator. Injury rates and RRs were expressed as the ratio of unweighted injury counts per 10,000 AEs. All other calculations, including injury proportion ratios (IPRs), used weighted data. The level of significance was α = 0.05 for all analyses.

An example of a RR calculation follows:

$$ \mathrm{RR}\;=\;\lbrack(\mathrm{Number}\;\mathrm{of}\;\mathrm{acute}\;\mathrm{injuries}\;\mathrm{in}\;\mathrm{2006-07}\;\mathrm{school}\;\mathrm{year}\;/\;\mathrm{Number}\;\mathrm{of}\;\mathrm{AEs}\;\mathrm{in}\;\mathrm{2006-07}\;\mathrm{school}\;\mathrm{year})\;\mathrm x\;\mathrm{10000}\rbrack\;\div\;\lbrack(\mathrm{Number}\;\mathrm{of}\;\mathrm{overuse}\;\mathrm{injuries}\;\mathrm{in}\;\mathrm{2006-07}\;\mathrm{school}\;\mathrm{year}\;/\;\mathrm{Number}\;\mathrm{of}\;\mathrm{AEs}\;\mathrm{in}\;\mathrm{2006-07}\;\mathrm{school}\;\mathrm{year})\;\mathrm x\;\mathrm{10000}\rbrack$$

An example of an IPR calculation follows:

$$ \mathrm{IPR}\;=\;\lbrack(\mathrm{Number}\;\mathrm{of}\;\mathrm{acute}\;\text{boys'}\;\mathrm{football}\;\mathrm{injuries}\;\mathrm{resulting}\;\mathrm{in}\;\mathrm{medical}\;\mathrm{disqualification}\;/\;\mathrm{Total}\;\mathrm{number}\;\mathrm{of}\;\mathrm{acute}\;\text{boys'}\;\mathrm{football}\;\mathrm{injuries})\;\mathrm x\;\mathrm{100}\rbrack\;\div\;[(\mathrm{Number}\;\mathrm{of}\;\mathrm{overuse}\;\text{boys'}\;\mathrm{football}\;\mathrm{injuries}\;\mathrm{resulting}\;\mathrm{in}\;\mathrm{medical}\;\mathrm{disqualification}\;/\;\mathrm{Total}\;\mathrm{number}\;\mathrm{of}\;\mathrm{overuse}\;\text{boys'}\;\mathrm{football}\;\mathrm{injuries})\;\mathrm x\;\mathrm{100}\rbrack$$

## Results

### Injury Frequency and National Estimates

From the 2006-07 through 2018-19 school year, 52 285 injuries were reported, which represent an estimated 17 434 646 injuries (95 % CI: 17 311 184–17 558 108) among high school athletes nationally, who participated in the 9 sports included in this study (Table 1). Most (92.0 %, *n* = 16 039 385) of these injuries were acute, while the remaining 8.0 % were overuse (*n* = 1 395 261). Overall, male athletes sustained 69.8 % of acute, 55.9 % of overuse, and 68.7 % of total injuries. However, for the gender-comparable sports, female athletes accounted for most acute (54.6 %), overuse (56.1 %), and total (54.8 %) injuries.

**Table 1:** Reported Number and National Estimates of Acute and Overuse Injuries by Year, Gender, Sport, and Exposure Type

Variable	Acute Injuries		Overuse Injuries		Total Injuries		*P*-value^b^
	RIO Data	National Estimate	RIO Data	National Estimate	RIO Data	National Estimate	
	n	n (%^a^)	n	n (%^a^)	n	n (%^a^)	
Total	48 585	16 039 385 (100.0)	3 700	1 395 261 (100.0)	52 285	17 434 646 (100.0)	
Year							< 0.0001
2006-07	4 154	1 286 717 (8.0)	385	139 393 (10.0)	4 539	1 426 111 (8.2)	
2007-08	4 323	1 276 352 (8.0)	343	111 132 (8.0)	4 666	1 387 484 (8.0)	
2008-09	3 848	1 105 534 (6.9)	287	109 887 (7.9)	4 135	1 215 421 (7.0)	
2009-10	3 386	1 229 912 (7.7)	235	108 970 (7.8)	3 621	1 338 883 (7.7)	
2010-11	3 271	1 110 073 (6.9)	230	89 924 (6.4)	3 501	1 199 997 (6.9)	
2011-12	3 552	1 307 022 (8.1)	243	103 851 (7.4)	3 795	1 410 873 (8.1)	
2012-13	3 925	1 332 860 (8.3)	220	79 594 (5.7)	4 145	1 412 454 (8.1)	
2013-14	3 899	1 359 033 (8.5)	271	94 574 (6.8)	4 170	1 453 607 (8.3)	
2014-15	3 557	1 162 379 (7.2)	207	69 853 (5.0)	3 764	1 232 232 (7.1)	
2015-16	3 876	1 305 912 (8.1)	336	120 217 (8.6)	4 212	1 426 129 (8.2)	
2016-17	3 190	1 085 989 (6.8)	234	95 192 (6.8)	3 424	1 181 180 (6.8)	
2017-18	3 850	1 266 419 (7.9)	374	139 610 (10.0)	4 224	1 406 028 (8.1)	
2018-19	3 754	1 211 182 (7.6)	335	133 063 (9.5)	4 089	1 344 245 (7.7)	
Gender (all sports)							< 0.0001
Male	36 548	11 192 580 (69.8)	2 239	780 359 (55.9)	38 787	11 972 939 (68.7)	
Female	12 037	4 846 805 (30.2)	1 461	614 902 (44.1)	13 498	5 461 707 (31.3)	
Gender (comparable sports)^c^							0.325
Male	9 266	3 461 432 (45.4)	996	398 349 (43.9)	10 262	3 859 781 (45.2)	
Female	9 781	4 166 279 (54.6)	1 142	508 419 (56.1)	10 923	4 674 699 (54.8)	
Sport							< 0.0001
Boys’ Football	23 584	6 748 875 (42.1)	1 051	331 029 (23.7)	24 635	7 079 904 (40.6)	
Boys’ Soccer	3 663	1 894 538 (11.8)	379	209 001 (15.0)	4 042	2 103 539 (12.1)	
Boys’ Basketball	3 912	1 025 551 (6.4)	267	77 135 (5.5)	4 179	1 102 685 (6.3)	
Boys’ Wrestling	3 835	1 011 042 (6.3)	192	49 484 (3.5)	4 027	1 060 527 (6.1)	
Boys’ Baseball	1 691	541 343 (3.4)	350	112 213 (8.0)	2 041	653 556 (3.7)	
Girls’ Soccer	4 456	2 497 513 (15.6)	452	270 186 (19.4)	4 908	2 767 699 (15.9)	
Girls’ Volleyball	2 119	651 757 (4.1)	319	107 979 (7.7)	2 438	759 736 (4.4)	
Girls’ Basketball	3 687	986 724 (6.2)	378	101 243 (7.3)	4 065	1 087 968 (6.2)	
Girls’ Softball	1 638	682 042 (4.3)	312	136 990 (9.8)	1 950	819 032 (4.7)	
Exposure Type^d^							< 0.0001
Competition	28 355	9 647 277 (60.2)	886	338 409 (24.3)	29 241	9 985 687 (57.3)	
Practice	20 219	6 390 158 (39.8)	2 814	1 056 852 (75.7)	23 033	7 447 010 (42.7)	

^a^ Percentages may not equal 100.0 % due to rounding

^b^*P*-values were based on Pearson chi-square comparing the frequencies of national estimates

^c^ Comparable sports are boys’ and girls’ soccer, boys’ and girls’ basketball, boys’ baseball and girls’ softball

^d^ Exposure types “Other” (n = 10) and “Performance” (n = 1) were excluded from p-value analysis

While boys’ football accounted for the most acute (42.1 %) and overuse (23.7 %) injuries among the 9 sports, boys’ baseball contributed the fewest (3.4 %) acute injuries and boys’ wrestling the fewest overuse injuries (3.5 %). Among female sports, girls’ soccer accounted for the most acute (15.6 % of all acute injuries) and overuse (19.4 % of all overuse injuries) injuries, while girls’ volleyball accounted for the least acute injuries (4.1 % of all acute injuries) and girls’ basketball the least overuse injuries (7.3 % of all overuse injuries).

The number of acute injuries increased slightly with increasing number of years in high school, with 23.3 % of acute injuries occurring among freshmen, 25.3 % among sophomores,

25.4 % among juniors, and 26.0 % among seniors; however, this increase was not statistically significant (p > 0.05). The number of overuse injuries showed no relationship with year in school, with 25.8 %, 24.5 %, 25.4 %, and 24.3 % occurring in freshman through senior years, respectively.

### Body Site and Diagnosis of Injuries

**Fig. 2:**
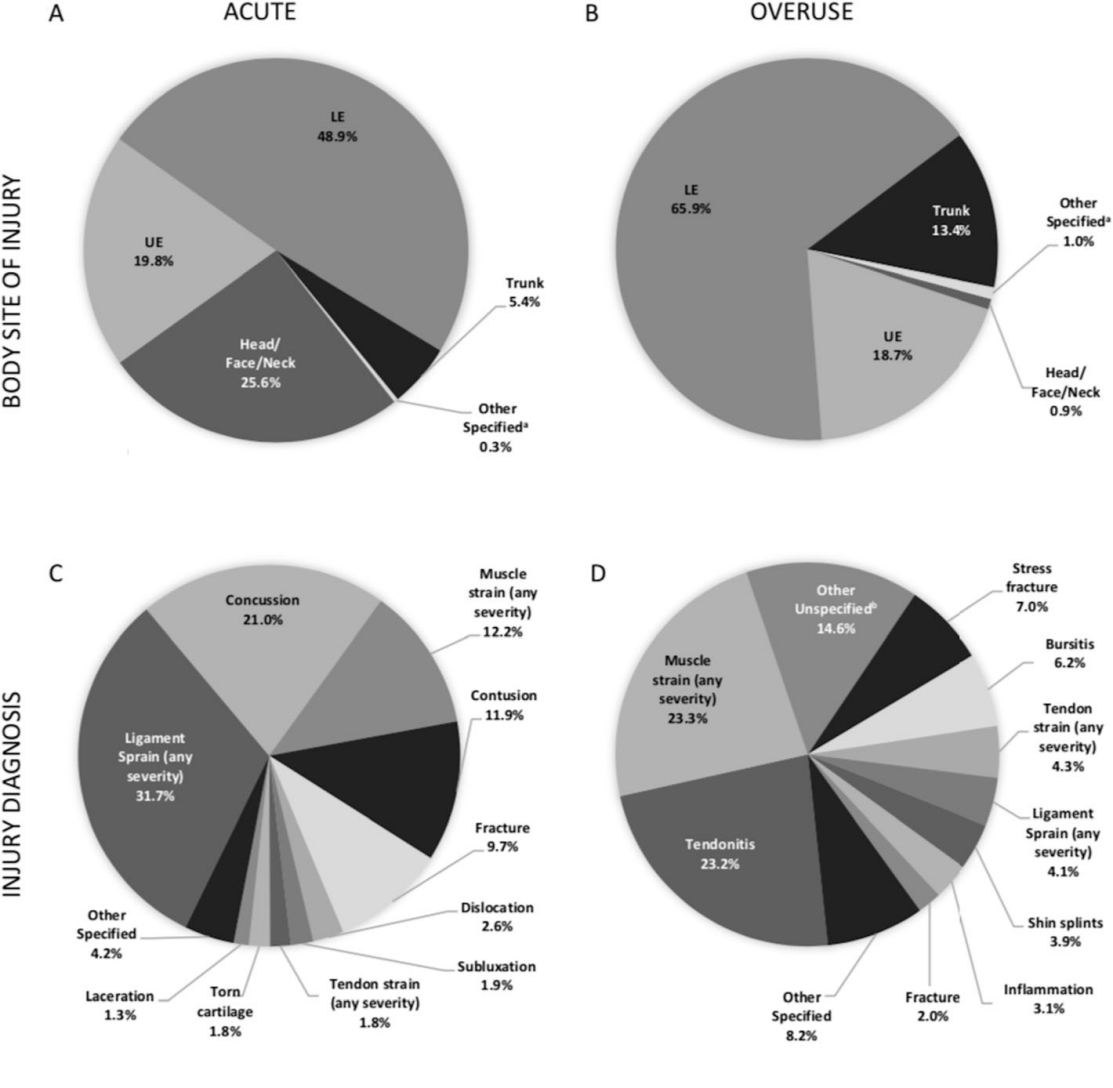
Body Site and Diagnosis of Acute and Overuse Injuries. **A** shows the body sites of acute injuries. **B** shows the body sites of overuse injuries. ^a^“Other specified” indicates injuries coded as “other” and “male genitalia”. **C** shows the top 10 diagnoses of acute injuries, with remaining diagnoses shown as “other specified”. **D** shows the top 10 diagnoses of overuse injuries, with remaining diagnoses shown as “other specified”. ^b^See methods section for description of “other unspecified”

Most acute (48.9 %) and overuse injuries (65.9 %) were to the lower extremities. The head/face/neck accounted for 25.6 % of acute injuries and 0.9 % of overuse injuries (Fig. 2 A and 2B). Among acute injuries, ligament sprains were the most common (31.7 %), followed by concussions (21.0 %), muscle strains (12.2 %), and contusions (11.9 %) (Fig. 2 C). Among overuse injuries, muscle strains (23.3 %) and tendonitis (23.2 %) were the most common (Fig. 2D).

### Injury Rates and Rate Ratios

Overall, the rate of acute injury (19.2 per 10,000 AEs) was approximately 13 times (RR: 13.12, 95 % CI: 12.69–13.57) higher than the rate of overuse injury (1.5 per 10,000 AEs) (Table 2). When comparing gender differences across all 9 sports, males had a higher rate of acute injury (23.6 per 10,000 AEs) than females (14.9 per 10,000 AEs) (RR: 1.58, 95 % CI: 1.54–1.61) and females had a higher rate of overuse injury (1.8 per 10,000 AEs) than males (1.4 per 10,000 AEs) (RR: 1.28, 95 % CI: 1.19–1.36). Male athletes were about 16 times (RR: 16.38, 95 % CI: 15.70–17.10) and female athletes were about 8 times (RR: 8.14, 95 % CI: 7.71–8.60) more likely to sustain an acute injury than an overuse injury. However, when comparisons were made within gender-comparable sports, female athletes had a higher rate of both acute (16.8 per 10,000 AEs; RR: 1.33, 95 % CI: 1.29–1.36) and overuse (2.0 per 10,000 AEs; RR: 1.44, 95 % CI: 1.32–1.57) injuries than males (acute 12.7 and overuse 1.4 per 10,000 AEs), and the likelihood of acute injury to overuse injury among male athletes (RR: 9.30, 95 % CI: 8.71–9.93) and female athletes (RR: 8.56, 95 % CI: 8.05–9.10) was similar.

**Table 2:** Injury Rates and Rate Ratios of Acute versus Overuse Injuries by Year, Gender, Sport, and Exposure Type

		Acute Injuries		Overuse Injuries		Injury Rate Ratio (95 % CI)
	AEs	Number	Rate per 10,000 AEs	Number	Rate per 10,000 AEs	
Variable						
Total	25 261 047	48 585	19.2	3 700	1.5	13.12 (12.69, 13.57)
Year						
2006-07	1 820 367	4 154	22.8	385	2.1	10.79 (9.72, 11.98)
2007-08	2 077 780	4 323	20.8	343	1.7	12.60 (11.29, 14.07)
2008-09	2 112 479	3 848	18.2	287	1.4	13.41 (11.89, 15.12)
2009-10	1 763 241	3 386	19.2	235	1.3	14.41 (12.62, 16.45)
2010-11	1 762 485	3 271	18.6	230	1.3	14.22 (12.44, 16.26)
2011-12	1 733 895	3 552	20.5	243	1.4	14.62 (12.84, 16.65)
2012-13	1 874 256	3 925	20.9	220	1.2	17.84 (15.58, 20.44)
2013-14	1 873 729	3 899	20.8	271	1.5	14.39 (12.72, 16.27)
2014-15	1 723 204	3 557	20.6	207	1.2	17.18 (14.94, 19.77)
2015-16	1 779 940	3 876	21.8	336	1.9	11.54 (10.32, 12.90)
2016-17	1 602 904	3 190	19.9	234	1.5	13.63 (11.94, 15.57)
2017-18	1 674 494	3 850	23.0	374	2.2	10.29 (9.26, 11.45)
2018-19	1 732 499	3 754	21.6	335	1.9	11.17 (9.99, 12.49)
Gender (all sports)						
Male	15 566 184	36 685	23.6	2 239	1.4	16.38 (15.70, 17.10)
Female	7 965 090	11 900	14.9	1 461	1.8	8.14 (7.71, 8.60)
Gender (comparable sports)						
Male	7 313 633	9 266	12.7	996	1.4	9.30 (8.71, 9.93)
Female	5 819 622	9 781	16.8	1 142	2.0	8.56 (8.05, 9.10)
Boys’ Sports						
Football	6 317 503	23 584	37.3	1 051	1.7	22.43 (21.09, 23.86)
Soccer	2 298 919	3 663	15.9	379	1.7	9.66 (8.70, 10.74)
Basketball	2 806 929	3 912	13.9	267	1.0	14.65 (12.94, 16.59)
Wrestling	1 935 048	3 835	19.8	192	1.0	19.97 (17.28, 23.09)
Baseball	2 207 785	1 691	7.7	350	1.6	4.83 (4.31, 5.42)
Girls’ Sports						
Soccer	2 001 410	4 456	22.3	452	2.3	9.85 (8.95, 10.86)
Volleyball	2 145 468	2 119	9.9	319	1.5	6.64 (5.90, 7.47)
Basketball	2 189 892	3 687	16.8	378	1.7	9.75 (8.77, 10.84)
Softball	1 628 320	1 638	10.1	312	1.9	5.25 (4.65, 5.93)
Exposure Type						
Competition	6 472 178	28 355	43.8	886	1.4	32.00 (29.93, 34.22)
Practice	17 061 395	20 219	11.9	2 814	1.7	7.19 (6.91, 7.47)

When comparing all 9 sports, boys’ football had the highest rate (37.3 per 10,000 AEs) and boys’ baseball had the lowest rate of acute injuries (7.7 per 10,000 AEs), while girls’ soccer had the highest rate (2.3 per 10,000 AEs) and both boys’ basketball and boy’s wrestling had the lowest rate of overuse injuries (1.0 per 10,000 AEs each). Compared with overuse injuries, the likelihood of acute injuries was highest for boys’ football (RR: 22.43, 95 % CI: 21.09–23.86), followed by boy’s wrestling (RR: 19.97, 95 % CI: 17.28–23.09) and boys’ basketball (RR: 14.65, 95 % CI: 12.94–16.59).

When comparing only the four female sports, soccer had the highest rate of acute injury (22.3 per 10,000 AEs) and highest RR of acute to overuse injury (RR: 9.85, 95 % CI: 8.95–10.86). Volleyball had the lowest rates of acute (9.9 per 10,000 AEs) and overuse (1.5 per 10,000 AEs) injury, and softball had the lowest RR of acute to overuse injury (RR: 5.25, 95 % CI: 4.65–5.93).

The rate of acute injury per 10,000 AEs was higher during competition (43.8) than practice (11.9) (RR: 3.70, 95 % CI: 3.63–3.76), while practice had a higher rate of overuse injury (1.7) than competition (1.4) (RR: 1.20, 95 % CI: 1.11–1.25). The risk of acute injuries compared with that of overuse injuries was higher during competition (RR: 32.00, 95 % CI: 29.93–34.22) than during practice (RR: 7.19, 95 % CI: 6.91–7.47).

### New Versus Recurrent Injuries

Most (90.3 %) acute injuries were new injuries, while 81.6 % of overuse injuries were new (IPR: 1.11, 95 % CI: 1.09–1.13). Recurrent injuries accounted for 9.7 % of acute injuries, while 18.6 % of overuse injuries were recurrent (IPR: 0.52, 95 % CI: 0.47–0.57).

### Time Loss

Compared with overuse injuries, acute injuries were less likely to result in time loss from sports participation of < 1 week overall (IPR: 0.70, 95 % CI: 0.68–0.74). This finding held true for both boys and girls and for all sports, except boys’ baseball (IPR: 0.94, 95 % CI: 0.81–1.10) (Table 3). Overall, acute injuries were more likely than overuse injuries to cause time loss of 1–3 weeks (IPR: 1.25, 95 % CI: 1.16–1.34), > 3 weeks (IPR: 1.30, 95 % CI: 1.10–1.52), or medical disqualification (IPR: 1.75, 95 % CI: 1.43–2.14). This was also true among both male and female athletes, except males for > 3 weeks of time loss (IPR: 1.10, 95 % CI: 0.92–1.32). When considering only gender-comparable sports, female athletes were more likely to be medically disqualified due to an acute versus an overuse injury (IPR: 2.43, 95 % CI: 1.59–3.72), while males demonstrated no difference in the proportion of medical disqualification (IPR: 1.51, 95 % CI: 1.00-2.28) from acute versus overuse injury.

**Table 3:** Time Loss Attributable to Acute and Overuse Injuries by Gender and Sport

	Time Lost to Acute Injuries					Time Lost to Overuse Injuries					Injury Proportion Ratios^c^				
	< 1 wk	1–3 wks	> 3 wks	Med DQ^a^	Other^b^	< 1 wk	1–3 wks	> 3 wks	Med DQ^a^	Other^b^	< 1 wk	1–3 wks	> 3 wks	Med DQ^a^	Other^b^
Variable	%^d^	%^d^	%^d^	%^d^	%^d^	%^d^	%^d^	%^d^	%^d^	%^d^	Ratio (95 % CI)	Ratio (95 % CI)	Ratio (95 % CI)	Ratio (95 % CI)	Ratio (95 % CI)
Total	38.9	35.5	8.1	5.6	11.8	55.1	28.4	6.3	3.2	7.0	**0.70 (0.68, 0.74)**	**1.25 (1.16, 1.34)**	**1.30 (1.10, 1.52)**	**1.75 (1.43, 2.14)**	**1.68 (1.45, 1.96)**
Gender (all sports)															
Male	39.3	34.7	8.4	5.8	11.8	52.3	28.8	7.7	4.0	7.3	**0.75 (0.71, 0.79)**	**1.20 (1.11, 1.31)**	1.10 (0.92, 1.32)	**1.46 (1.16, 1.83)**	**1.61 (1.33, 1.95)**
Female	37.9	37.5	7.4	5.2	11.9	58.8	27.9	4.5	2.2	6.6	**0.65 (0.60, 0.69)**	**1.35 (1.20, 1.51)**	**1.65 (1.23, 2.23)**	**2.33 (1.56, 3.50)**	**1.80 (1.40, 2.31)**
Gender (Gender- comparable sports)															
Male	43.7	34.1	7.5	4.4	10.3	54.7	26.8	7.8	2.9	7.8	**0.80 (0.74, 0.86)**	**1.27 (1.11, 1.46)**	0.96 (0.72, 1.27)	1.51 (1.00, 2.28)	1.32 (0.99, 1.75)
Female	36.9	37.7	7.4	5.7	12.4	57.6	28.9	4.8	2.3	6.4	**0.64 (0.59, 0.69)**	**1.31 (1.15, 1.49)**	**1.54 (1.11, 2.13)**	**2.43 (1.59, 3.72)**	**1.93 (1.44, 2.57)**
Sport															
Boys’ Football	38.2	34.9	8.5	6.2	12.2	48.7	31.4	8.3	4.8	6.7	**0.78 (0.73, 0.85)**	1.11 (0.99, 1.24)	1.02 (0.81, 1.30)	1.29 (0.95, 1.75)	**1.82 (1.38, 2.39)**
Boys’ Soccer	44.1	34.0	6.7	4.3	10.9	61.1	23.8	6.4	2.4	6.3	**0.72 (0.65, 0.81)**	**1.43 (1.14, 1.79)**	1.05 (0.63, 1.77)	1.78 (0.87, 3.65)	**1.73 (1.03, 2.92)**
Boys’ Basketball	44.3	35.5	7.6	3.9	8.7	53.1	26.6	7.7	3.4	9.2	**0.83 (0.73, 0.96)**	**1.33 (1.05, 1.69)**	0.99 (0.60, 1.63)	1.16 (0.56, 2.40)	0.95 (0.59, 1.53)
Boys’ Wrestling	32.0	35.3	11.4	7.3	14.0	56.7	27.0	2.4	6.6	7.2	**0.56 (0.48, 0.67)**	1.30 (0.96, 1.77)	**4.78 (2.49, 9.17)**	1.16 (0.56, 2.40)	**1.94 (1.07, 3.50)**
Boys’ Baseball	41.2	32.1	10.0	5.4	11.2	43.7	32.5	10.6	3.4	9.7	0.94 (0.81, 1.10)	0.99 (0.81, 1.21)	0.94 (0.63, 1.41)	1.57 (0.85, 2.91)	1.15 (0.77, 1.72)
Girls’ Soccer	35.6	38.5	6.8	5.7	13.4	56.7	30.4	4.1	2.6	6.2	**0.63 (0.56, 0.71)**	**1.27 (1.04, 1.54)**	1.67 (0.95, 2.93)	**2.18 (1.21, 3.90)**	**2.16 (1.37, 3.40)**
Girls’ Volleyball	44.8	36.1	7.6	2.6	9.0	64.4	23.1	3.0	1.9	7.6	**0.70 (0.62, 0.78)**	**1.56 (1.20, 2.02)**	**2.54 (1.25, 5.19)**	1.37 (0.40, 4.68)	1.17 (0.72, 1.91)
Girls’ Basketball	38.2	37.8	8.0	5.9	10.1	55.9	26.1	8.7	2.1	7.3	**0.68 (0.61, 0.77)**	**1.45 (1.18, 1.78)**	0.93 (0.61, 1.39)	**2.85 (1.35, 5.98)**	1.39 (0.90, 2.15)
Girls’ Softball	39.6	34.9	8.6	5.1	11.9	60.4	28.0	3.4	1.9	6.3	**0.66 (0.57, 0.75)**	1.24 (0.99, 1.57)	**2.53 (1.23, 5.19)**	**2.64 (1.05, 6.62)**	**1.90 (1.11, 3.25)**

^a^ Includes Medical Disqualification (Med DQ) for season or career

^b^ Includes Athlete chooses not to continue, Athlete released from team, Other, and Season ended before athlete returned to activity

^c^ Injury Proportion Ratios = Acute / Overuse. Formula is included in the methods section. Significant Ratios appear in bold

^d^ Percentages represent row percentages and may not sum to 100.0 % because of rounding

Among all sports, the likelihood of medical disqualification from acute injury was higher than that from overuse injury for girls’ basketball (IPR: 2.85, 95 % CI: 1.35–5.98), girls’ softball (IPR: 2.64, 95 % CI: 1.05–6.62), and girls’ soccer (IPR: 2.18, 95 % CI: 1.21–3.90). All other sports demonstrated no difference in the proportion of medical disqualification from acute versus overuse injury.

## Discussion

This is the first study to compare acute and overuse injuries using a nationally representative sample of high school athletes participating in 5 boys’ sports (football, soccer, basketball, wrestling, baseball) and 4 girls’ sports (soccer, volleyball, basketball, softball). There was an average of 1 341 127 high school sports-related injuries per year, equating to an average of 153 injuries every hour, associated with the sports included in this study. In addition to the monetary toll an injury may take, which can be upwards of $700 per injury in direct medical costs, athletes can be subject to long-term physical and psychological sequelae (Knowles et al. 2007; Maffulli et al. 2010; Shuer and Dietrich 1997). The consequences of injury, when combined with the frequency of injury and rising sport participation numbers, represent a large burden on both the student athletes’ health and the health care system.

Prior studies of collegiate and high school athletes have shown that females are at higher risk of overuse injury than males, with possible reasons cited as gender differences in biomechanics, coaching, or help-seeking behavior (Yang et al. 2012; Post et al. 2020; Roos et al. 2015). In concordance with this, when considering all 9 sports in this study, female athletes had a higher rate of overuse injury than males, while male athletes had a higher rate of acute injury. This was largely attributable to the disproportionately high rate of acute injury associated with football. When considering only gender-comparable sports, female athletes experienced higher rates of both acute and overuse injury than males, though males accounted for most acute and overuse injuries because of higher participation numbers. These findings underscore the potential benefit of implementation and evaluation of additional prevention measures in a gender- and sport-specific manner. In addition, acute injury prevention efforts in collision sports like football can have a large impact, where just a 1 % decrease in occurrence equates to almost 70,000 injuries avoided nationwide per year.

Time loss associated with acute and overuse injuries demonstrated important differences. Acute injuries were less likely to cause < 1 week of time loss than were overuse injuries for both male and female athletes. Acute injuries were more likely than overuse injuries to cause a time loss of 1–3 weeks or medical disqualification among both male and female athletes; this was also true among female (but not male) athletes for > 3 weeks of time loss. These observed differences are most likely a result of the nature of these types of injury; while overuse injuries are the result of repetitive microtrauma with inadequate rest time for repair, acute injuries can result from greater forces resulting in injuries that may require longer healing time (Roos et al. 2014; DiFiori et al. 2014; Hubbard et al. 2008; Karladani et al. 2001). These results may also be due to the number of concussive injuries which often require > 1 week of recovery time when following return-to-play protocols (Tamura et al. 2020). Although time loss due to some injuries may be inevitable, the development of training/competition schedules with adequate rest time built in and continued improvement of treatment protocols may lead to quicker return-to-play and reduce the number medical disqualifications for both acute and overuse injuries.

The lower extremities were the most common body site for both acute and overuse injuries in this study. While the sports included in this study may predispose to more injuries to the lower extremities than other body sites, this finding is in agreement with previous studies, which included swimming, gymnastics, and tennis in addition to many of the sports in the current study (Yang et al. 2012; Rechel et al. 2008; Roos et al. 2015). With approximately half of acute and two-thirds of overuse injuries occurring to the lower extremities, this has clear implications regarding where prevention efforts are needed. Acute injuries also commonly occurred in the head/face/neck region. This was largely attributable to concussions, which accounted for almost one-fourth of acute injuries. Sports-related concussions have been the focus of new laws and regulations during recent years. Although implementation of these laws led to increased recognition of concussions and therefore an increased reporting of concussions, preliminary research has shown a decline in the recurrent concussion rate within 3 years of law implementation that may be attributable to changes in return-to-play requirements (Yang et al. 2017). A similar public policy approach may help reduce other types of sports-related injury.

Prior research has reported that injury recurrence is more commonly attributable to an overuse mechanism, while new injuries are more likely a result of contact with another person (Welton et al. 2018). Our study was consistent with these findings, with a higher proportion (19 %) of overuse injuries being recurrent than among acute injuries (10 % were recurrent), and 90 % of acute injuries being new injuries compared with 82 % of overuse injuries being new. Previous studies have reported that an average of about 10 % of sports injuries in high school each year are recurrent, which is similar to the proportions of recurrent injury in our study; 19 % of overuse and 10 % of acute injuries were recurrent (Welton et al. 2018; Powell and Barber-Foss 1999). Although the mechanism of injury likely plays a role in injury recurrence, rehabilitation strategies may also be a factor. Acute injuries may have better-defined timelines for healing and return-to-play based on diagnosis or severity, whereas recovery from overuse injuries may be more subjective in nature. The prevalence of recurrent injury in high school sports offers an opportunity for improved treatment strategies and rehabilitation techniques to reduce the likelihood of an injury occurring more than once. Future research should explore the causes of recurrent injuries, for example, whether they are due to poor technique, risk-taking by the athlete, or incomplete healing; this would allow development of targeted interventions to help prevent these injuries.

The type of exposure greatly influenced the risk of acute versus overuse injury. Overall, during competition, athletes were 32 times more likely to sustain an acute injury than an overuse injury, while during practice, they were only 7 times more likely. A prior study showed that practice injuries occur at lower rates than competition injuries, and this was attributed to competition having an increased speed of play and an increased likelihood for more forceful collisions (Rechel et al. 2008). In our study, the acute injury rate during competition was almost four times that during practice, while overuse injuries occurred at similar rates regardless of the type of exposure. This demonstrates that the overall difference between competition and practice injury rates is driven by acute injury occurrence.

### Study Limitations and Strengths

This study has some limitations. Injuries must result in time loss from sports participation to be reported in RIO™ (except for fractures, concussions, dental injuries, and heat illness/injury); therefore, both acute and overuse injuries may be underreported because not all prevent an athlete from returning to play. Previous research showed that 50.8 % of overuse and 29.8 % of acute injuries among athletes from one NCAA Division 1 program resulted in no time loss (Yang et al. 2012). This issue may especially affect overuse injuries because they are progressive in nature. Additionally, overuse injuries may not be reported until a specific event causes time loss for an athlete, which may alter the categorization of these injuries to acute. Some sports that may predispose to more overuse injuries (track and field, swimming, etc.) were not included in this study because RIO™ only captures injuries associated with these sports using convenience sampling. Overall, the definition of overuse injury has, historically, not been clearly defined. This may limit comparison between our study and those of other investigators. The lack of exposure data in RIO™ for some variables, such as diagnosis, body site injured, and athlete’s year in school, precluded calculation of rates for these variables and is a limitation of this study. Despite these limitations, a strength of this study is that it utilized a large, nationally representative sample of injury and exposure data collected by ATs using strict data quality procedures. RIO™ data have been used in more than one hundred peer-reviewed publications to-date, attesting to the strength and quality of this surveillance system and database. To our knowledge, this is the first study to compare characteristics of acute and overuse injuries at the national level. This study provides a foundation for additional studies that better define and quantify overuse injuries or compare acute and overuse injuries and associated risk factors within specific sports.

## Conclusions

Acute injuries accounted for most high school sports-related injuries and occurred at a higher rate than overuse injuries in the 5 boys’ sports (football, soccer, basketball, wrestling, baseball) and 4 girls’ sports (soccer, volleyball, basketball, softball) studied. They also resulted in greater time loss from sports participation than overuse injuries and occurred at a much higher rate during competition than practice. Overuse injuries were more likely to be recurrent injuries than acute injuries, while acute injuries were more often new injuries. Attention to these differences between acute and overuse injuries can help inform improved athlete preparation (such as conditioning and strength training), treatment, rehabilitation, and schedules allowing for adequate rest time, which may reduce injuries and improve injury outcomes in high school athletics.

## Data Availability

Data analyzed in this study were from the High School RIO™ surveillance system, which is managed by the Datalys Center for Sports Injury Research and Prevention. Data requests should be submitted to the Datalys Center.

## Abbreviations

AE: Athlete Exposure
AT: Certified Athletic Trainer
CI: Confidence Interval
IPR: Injury Proportion Ratio
RIO: Reporting Information Online
RR: Rate Ratio
US: United States
